# Exploring Orthographic Representation in Chinese Handwriting: A Mega-Study Based on a Pedagogical Corpus of CFL Learners

**DOI:** 10.3389/fpsyg.2022.782345

**Published:** 2022-03-10

**Authors:** Jun Zhang

**Affiliations:** College of Advanced Chinese Training, Beijing Language and Culture University, Beijing, China

**Keywords:** handwriting, orthographic representation, logographeme, inter-stroke interval, pedagogical corpus

## Abstract

Writing and reading are closely related and are thus likely to have a common orthographic representation. A fundamental question in the literature on the production of written Chinese characters concerns the structure of orthographic representations. We report on a Chinese character handwriting pedagogical corpus involving a class of 22 persons, 232 composite character types, 1,913 tokens, and 13,057 stroke records, together with the inter-stroke interval (ISI), which reflects the parallel processing of multilevel orthographic representation during the writing execution, and 50 orthographic variables from the whole character, logographeme, and stroke. The results of regression analyses show that orthographic representation has a hierarchy and that different representational levels are active simultaneously. In the multilevel structure of orthographic representation, the representation of the logographeme is absolutely dominant. Writing and reading have both commonalities and individual differences in their orthographic representations. The online processing of the logographeme unit probably occurs at the ISI before the initial stroke of the current logographeme, which may also cascade to the first subsequent logographeme. In addition, we propose a new effective character structure unit for describing orthographic complexity.

## Introduction

Writing and reading are two forms of word processing. Writing is the process from concept preparation to outputting an orthographic representation, whereas reading is a process of accessing meaning from orthographic representation. In the process of Chinese character learning, writing and reading are often carried out synchronously and have a positive correlation with each other (Tan et al., 2005; Chan et al., 2006). Indeed, it has been shown that handwriting literacy affects reading literacy, such that both children who speak Chinese as their mother tongue who are better at handwriting and adult learners of Chinese as a foreign language (CFL) who are better at handwriting are also better at reading (Tan et al., 2005; Guan et al., 2015). Chinese character handwriting can promote the formation of long-term motor memory of Chinese characters and strengthen the orthographic representation of Chinese characters, which is conducive to extracting the visuospatial information required for Chinese character recognition. Moreover, the acquisition of Chinese characters by writing them activates more of the sensory-motor cortex and improves the accuracy of Chinese character recognition to a greater extent than does acquisition by writing *pinyin* (Cao et al., 2013). Therefore, reading and writing are likely to have a common orthographic representation. Studying how to represent Chinese characters in handwriting can help us gain a deeper understanding of the cognitive mechanisms underlying writing and Chinese character recognition in reading.

Handwriting includes not only the central processes underlying conceptual preparation, lexical selection, and orthographic access but also the peripheral processes of motor programming and the actual writing execution (Baxter and Warrington, 1986). According to van Galen’s (1991) model of handwriting production, handwriting is not just a succession of isolated acts but also rather an organized, hierarchical process in which the various representational levels are processed in parallel. The higher level of this model includes the intention to generate a message, the setting up of semantic information, lexical retrieval, and syntactic organization of the sentence, as in Levelt’s (1989) model of oral production. The lower levels of van Galen’s model are specific to the written modality and are concerned with the retrieval of spelling, selection of allographs, regulation of size control, and muscular adjustments. As all levels are activated in parallel during handwriting, high- and low-level modules operate at the same time. Because processing capacities are limited, this processing increases over the duration of the movement/writing execution. This means that the movements to write the forthcoming graphemes (i.e., strokes, letters, logographemes, or syllables) must be programmed during the execution of the initial graphemes. By comparing the distribution patterns of intervals between each two handwriting units at different levels (e.g., letter and syllable), researchers can infer the orthographic representational units and their content (Kandel et al., 2006, 2009; Kandel and Valdois, 2007; Vilageliu and Kandel, 2012). There are obvious interval time peaks among graphemes during handwriting, which implies the simultaneous processing of the spelling of the following grapheme and the local parameters, such as size and rotation direction, of the current grapheme.

Studies of the orthographic representation of Chinese character handwriting to date have employed a typical lab research set-up, where a small sample of native Chinese-speaking participants handwrite a small set of selected characters (Chen and Cherng, 2013; Damian and Qu, 2017), or are based on the systematic observation of native Chinese-speaking patients with brain injuries (Law and Leung, 2000; Han et al., 2007; Han and Bi, 2009). Although these studies have shed much light on the orthographic representations underlying handwriting, the extent to which findings from small numbers of healthy native Chinese speakers (or patients) and small stimulus samples are generalizable to CFL learners or to larger numbers of characters remain a topic of debate. Therefore, a large-scale pedagogical corpus of handwriting responses by CFL learners to a large sample of characters that are covered in the syllabus to explore the orthographic representation of character handwriting and to discuss the cognitive mechanisms underlying handwriting for both native speakers and CFL learners is needed. Chinese characters are complex things, and strokes are the smallest unit of handwriting. In this study, we investigate the inter-stroke interval (ISI) in a Chinese handwriting pedagogical database of learners of Chinese as a foreign language, looking at the contributions of the characteristics of orthographic units, to explore the orthographic representation of character handwriting.

## The Characteristics of Chinese Characters

Chinese is a logographic language. The basic writing units are Chinese characters (also called *Hanzi*), which usually correspond to a syllable in sound and a morpheme in meaning. There are more than 20,000 characters in the modern Chinese language, including about 3,000 commonly used characters (Han et al., 2007). Linguists generally analyze a character spatially on three levels: radical (Law, 2004), logographeme (Han et al., 2007), and stroke (Luo et al., 2008). For example, the character 清 (/qing1/, clear) is composed of two radicals (氵 and 青), three logographemes (氵, 龶, and 月), and eleven strokes (e.g.,丶, 一, 丨) in a two-dimensional space. The number of strokes and the number and position of radicals/logographemes have been shown to be important determinants of Chinese character naming (Leck et al., 1995; Peng and Wang, 1997; Peng et al., 2006) and have been shown to affect handwriting (Shi et al., 2011; Zhang and Feng, 2017). About 85% of *Hanzi* are pictophonetic characters (Zhu, 1988), which are composed of a semantic radical (e.g., 氵 in 清) that suggests the meaning of the character and a phonetic radical (青 in 清) that provides cues to its pronunciation. About 64% of radicals can be further divided into logographemes, which are the smallest units between radicals and strokes in a character that are spatially separated (State Language Commission, 1998; Law and Leung, 2000), for instance, 龶 and 月 in 清; the remaining radicals serve as a single logographeme.

Whereas in orthographically shallow languages (e.g., Spanish), the orthography–phonology correspondence is direct, there is no segmental correspondence between radicals/logographemes and the pronunciation of the character in Chinese (Han and Bi, 2009; Huang et al., 2021a). Pronounceable radicals/logographemes are characters themselves and account for approximately 60% of the characters listed in *Specification of Common Modern Chinese Character Components and Component Names* (现代常用字部件及部件名称规范; Ministry of Education of China, 2009). The transparence between orthography and phonology operates at the whole-syllable level by means of phonetic radicals and only applies to a subset of characters (Han and Bi, 2009). Although phonetic radicals are not very reliable indices, they have been shown to affect the processing of Chinese characters in reading and handwriting access (Law et al., 2005; Wang et al., 2019). Many studies have proposed that the syllable is the grapheme that acts as the handwriting motor unit in alphabetic languages (Kandel et al., 2006) and that different degrees of transparency affect “online” orthographic access in handwriting execution. In Chinese, the effect of the phonetic identity of radicals/logographemes in orthographic representation has been supported by the study of patients with brain injuries (Han and Bi, 2009), but some studies have suggested that this impact does not occur in the motor execution period (Wang et al., 2019; Huang et al., 2021b).

The writing of *Hanzi* follows established stroke order norms, such as “left before right,” “up before down,” “middle before both sides,” etc. (Ministry of Education of China, 2020). Skilled stroke order memory is helpful in activating the representation of characters (Flores d’Arcais, 1994). However, the stroke itself may be a combination of lines in multiple directions. There are 8 basic strokes which generate 29 compound strokes (Wang and Su, 2012). Related studies further subdivide strokes into *segment*s (also called “substrokes” or “strokemes”) from the perspective of graphics. For example, the second stroke 𠃌 in 句 (/ju4/, sentence) can be analyzed into three segments according to the turning point during the stroke trajectory (Ministry of Education of China, 2001). Although the segment is a graphic concept, using the segment as the primitive of Chinese characters can effectively improve the accuracy and efficiency of Chinese character computer recognition, and experiments show that the number of segments has an effect on orthographic access to Chinese characters (Wang et al., 2013).

## Orthographic Representation in the Handwriting Production of *Hanzi*

The complex structure of Chinese characters offers a rich research perspective for studying the orthographic representation of Chinese character handwriting. Law and Leung (2000) proposed that the logographeme constitutes the basic orthographic processing unit underlying Chinese written production, based on evidence from brain-impaired dysgraphic patients, because the type of writing error produced by the Cantonese-speaking patient in question, SFT, mostly took place at the level of the logographeme (211/315). Law and Leung’s (2000) argument was, however, subsequently questioned by Han et al. (2007) because the errors made in delayed copying and direct copying had accuracy rates of 40 and 53%, respectively, and the patient clearly performed much better on an oral than on a written naming task. Therefore, the damage to SFT was not in the grapheme buffer, but more likely at the peripheral stage. Han et al. (2007) reported the case of a brain-damaged Mandarin speaker, WLZ, who had not sustained a peripheral motor system injury, as direct copying was 100% correct. Delayed copy was not affected by lexicon variability and showed an obvious word-length effect. Most of the errors occurred at the logographeme level, and the greater the number of logographemes, the lower the accuracy of copying. Therefore, the existing evidence shows that the basic orthographic representation unit of *Hanzi* is a logographeme, which is stored in the *logographeme output buffer*, much like the *grapheme output buffer* proposed by Rapp and Caramazza (1997). In addition, Han et al. (2007) found that many response logographemes tend to have the same strokes as the target logographemes at levels significantly higher than random. Therefore, orthographic representation includes not only the identity of the logographeme itself but also the stroke and other information, which has been confirmed to a certain extent by Lo et al. (2016). Lo et al. (2016) examined the contribution of the types of orthographic knowledge to handwriting traditional *Hanzi* in a two-year longitudinal study and proposed that stroke knowledge makes a unique contribution to writing performance and has a non-significant effect on reading performance.

Han and Bi (2009) reported the case of a Mandarin-speaking individual suffering from a brain injury, MZG, who had normal auditory and visual comprehension and oral production and had not sustained a peripheral motor system injury, with near-perfect performance in oral reading (30/30), oral picture naming (29/30), delayed copying (28/30), and direct copying (30/30). Therefore, the patient preserved intact input processing, the conceptual system, and handwriting motor ability. He had, however, significant difficulty in written picture naming (19/30) and writing to dictation (17/30), indicating that MZG’s writing deficit should lie in the processing stages between lexical retrieval and motor execution, probably at the point of retrieval of the shapes of the character radicals/logographemes. Such patterns in the first Chinese-speaking case documenting an oral spelling preservation in the face of dysgraphia show that the phonological identities of radicals/logographemes are part of the orthographic representation of *Hanzi*.

Some evidence with regard to logographemes from healthy adults has also been reported by Chen and Cherng (2013), who used the implicit priming task to explore the relevant planning units of Chinese written production and found that the priming effect occurred when logographemes overlapped among responses. Furthermore, the authors introduced a model for Chinese handwritten character generation, which consists of two separate and serial stages of form encoding: “morphological encoding” and “orthographic encoding.” During orthographic encoding, the logographeme as a “proximate unit” of handwriting (O’Seaghdha et al., 2010) is specified and associated with a structural frame according to the orthotactic principles of written Chinese. However, Chen and Cherng’s (2013) experimental results were not replicated by Damian and Qu (2017). The latter study used similar experimental paradigms and found strong evidence for radical-based effects but only weak evidence for logographemic priming effects. The authors provided possible reasons for this discrepancy in terms of the potential differences between simplified and traditional script.

In addition to the above two research paradigms, analyzing the types of writing errors or the latency before writing execution, Zhang and Feng (2017) designed two delayed-copy *Hanzi* experiments to test the effects of component (radical or logographeme), complexity (few or many strokes), stroke position (radical boundary or non-radical boundary stroke), and lexicality (character or non-character) on two dependent variables: the latency (as the index of central processing) and stroke velocity (as the index of peripheral processing) of correct handwritten forms. The findings showed that, regardless of lexicality, writing latencies were longer for characters with higher complexity than for characters with lower complexity, indicating that the adult participants needed to prepare the whole character before initiating writing execution. The velocities of the strokes at the radicals’ boundaries were slowest, indicating that there is a radical boundary effect in writing execution due to the “online” planning of a second radical within a character and the fact that radicals/logographemes are a processing unit in Chinese. Interestingly, both lexicality and radical complexity affected the central processing and cascaded over peripheral processing during the execution of *Hanzi* writing.

To our knowledge, the only research to examine CFL learners with respect to orthographic representation in Chinese character handwriting production is that reported by Xu et al. (2018). According to Kandel et al. (2006), these researchers adopted digital ink technology and designed a lab experiment with three factors: Chinese level, critical stroke position, and *Hanzi* structure; the dependent variable was the critical ISI in the writing process. The experiment assumed that the processing retrieval time of the same stroke in different positions (between logographemes or inside logographemes) is different. For example, the third stroke “丿” is located inside the first logographeme (穴 in 窄) but is a starting stroke in the second logographeme (谷 in 容). The 36 participants were required to copy 24 pairs of orthographically similar characters, and the results showed that the critical ISI between the logographemes was longer than within a logographeme. This is consistent with most of the above studies of native writers, which suggest that there is a logographeme boundary effect in Chinese characters with up–down structures and left–right structures. Although the study proposed that logographemes are the units of representation of orthography, the experiment only compared the difference in ISI before a critical stroke in each pair of orthographically similar characters; that is, it only checked the effect of the identity of the logographeme where the critical stroke was located on its ISI. It was impossible to determine whether there was also a logographeme boundary effect between other logographemes in multi-logographeme *Hanzi*. Logically, if a character unit smaller than a logographeme is a unit of representation, it is reasonable to have a logographeme boundary effect. Therefore, it is necessary to comprehensively investigate the effect of other character units on ISI, including critical strokes and non-critical strokes. In addition, this study also had the limitations of previous studies, and the stimulus sample was small. In the present study, we will continue to take ISI as the index of parallel processing of multilevel orthographic representation and conduct a detailed study of the depth and breadth of orthographic representation in a large-scale pedagogical handwriting database of CFL learners.

## The *Hanzi* Handwriting Pedagogical Corpus of Chinese as a Foreign Language Learners

### Participants

This database was established under a conventional teaching plan, including direct copy handwriting datum in a specific range of *Hanzi* types. One class of 22 CFL adult learners (14 males, 8 females; age range = 18–30 years with a mean of 21.5) in Beijing Language and Culture University was the source of data collection. These participants were from 12 countries from non-Chinese-character cultural circles, namely Russia, Kyrgyzstan, Tajikistan, Kazakhstan, Mongolia, Italy, Switzerland, Macedonia, Bulgaria, Guinea, Indonesia, and Morocco. Their native languages were very diverse, including nine languages, such as Russian, Mongolian, Italian, Bulgarian, and French, which may have minimized the possibility of this research being systematically affected by mother tongue factors. This was related to the class division measures of the college, as the university managers hoped that learners at the same level would be in a multicultural learning environment to reduce their dependence on their mother tongue. The participants were all total beginner learners and had no experience of Chinese education before taking part in the study. Limited by the implementation of teaching, although the sample of participants is small, which may undermine the validity of the results, the subject group can still be regarded, to a certain extent, as a CFL beginner sample with a high diversity of mother tongue backgrounds and a high degree of homogenization of Chinese language level. Before the establishment of the corpus, the students were told that the performance of their direct copy handwriting tasks in class would be recorded in their semester results. All had normal or corrected-to-normal vision and intact reading and handwriting ability.

### Materials

All *Hanzi* types came from the first three volumes of *Road to Success* (Qiu, 2010), the official teaching materials for teaching college Chinese in the first one and a half months of the semester. During this period, students study *Hanzi* knowledge intensively and learn how to write under special *Hanzi* teaching guidance. We collected 232 types and 1,913 tokens of composite *Hanzi*, in which the average number of segments, strokes, and logographemes of characters in the database are 10.96 (*SD* = 3.07), 8.69 (*SD* = 2.64), and 2.6 (*SD* = 0.76), respectively, taking ISI as the observation index, and looked at the contributions of different orthographic units’ characteristics. Subsequently, we decomposed each composite token and stored data records according to the number of strokes and stroke order. For example, the character 男 (/nan2/, male) can be decomposed into seven strokes: 丨, 𠃍, 一, 丨, 一, 𠃌, 丿. Except for the first stroke of every *Hanzi*, there is an ISI before the other remaining strokes, which was used to characterize the “online” processing during handwriting execution. Then, 1,913 composite characters were decomposed into 14,970 stroke records, including 13,057 stroke records with ISI information and 1,913 stroke records (first stroke status) lacking ISI information.

### Apparatus and Procedure

**FIGURE 1:**
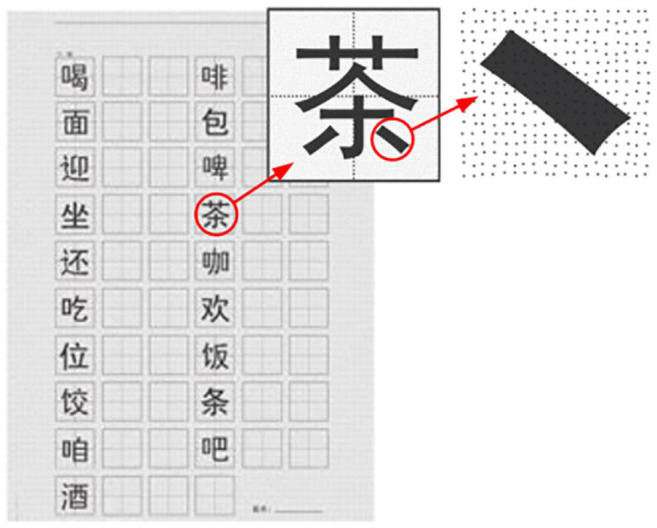
Direct copy task.

**FIGURE 2:**
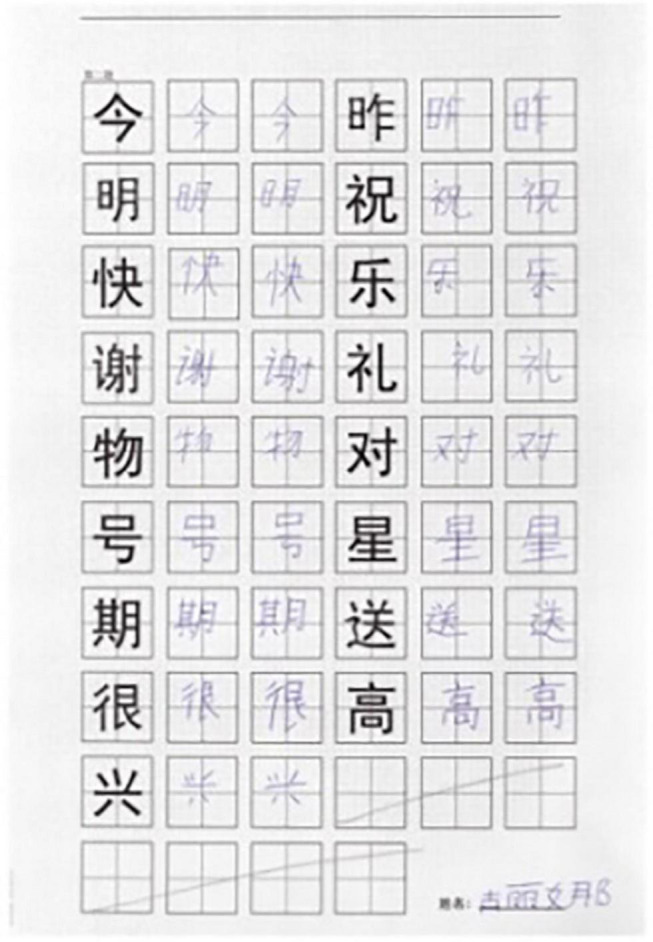
Handwriting sample.

Each direct copying task was incorporated into the normal teaching procedure. As the students had been taught the writing knowledge of the target Chinese characters, such as strokes, logographemes, and stroke orders, before the task in the same class, it was assumed that all participants would have the same amount of Chinese character writing knowledge. The specific requirements of the copying task were: The target character was printed directly on the paper, which was full of small points. All of the points formed a dot matrix to construct a two-dimensional space with coordinate information, as shown in Figure 1. The participants were required to start to follow the target character and to handwrite it twice with an Anoto Digital Pen at the same time in class until the copy task was completed (up to 10 min) or the students terminated in advance by themselves, as shown in Figure 2. In the process of copying, the students were allowed to look back at the target Chinese characters freely. They could, however, only write the character twice, so the students always wrote as much as possible and showed their handwriting ability.

**FIGURE 3:**
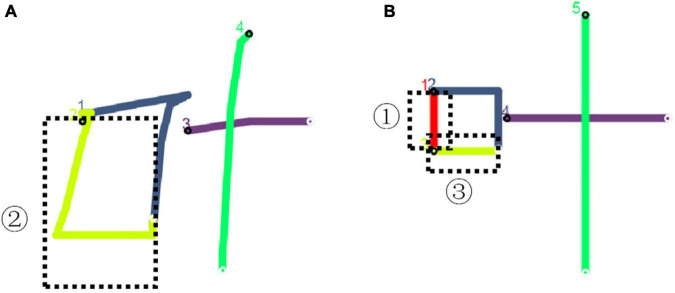
**(A, B)** The visualization of “叶” written by a writer and the standard form.

The digital pen recorded the digital ink data of the handwriting of Chinese characters by sensing the pressure formed by the contact between the pen and the paper. In the handwriting movement, the process from making a stroke to lifting the pen is a *stroke-step* (*SS*). The pen tip is suspended between two *SS*s, and the pressure is 0. When a writer writes Chinese characters in a standardized stroke form according to the stroke order criterion, the *SS* corresponds to the stroke. On the contrary, there is no corresponding relationship between the two. For example, the first and third strokes of “叶” in standard form should be written as “丨” and “―,” respectively, as shown in Figure 3B in the black dotted box. One writer may put these two strokes together by mistake and finish them in one *SS* like “𠃊,” as shown in Figure 3A. In order to eliminate the pollution of the dependent variable *ISI* caused by stroke shape or order errors, this study deleted the 3,191 wrong tokens and only retained the 1,913 correct tokens as analytical materials.

#### Orthographic Variables From the Whole Character, Logographeme, and Stroke Character Composition and Complexity

We used the *Dictionary of Chinese Character Information* (汉字信息字典; Chinese Character Encoding Group of Shanghai Jiao Tong University, and Chinese Pinyin Characters Research Group in Shanghai, 1988) to determine the characters’ compositions. In particular, a character was coded as having a left–right composition if its radicals are horizontally arranged, a top–down composition if the radicals are vertically arranged, or a surrounding composition if its radicals are surrounded by other radicals in whole or in part. Because character composition is a categorical variable, it was dummy coded with three variables: “*ChaLR*” refers to whether a character has a left–right composition (labeled 1) or not (labeled 0), “*ChaUD*” refers to whether a character has an up–down composition (labeled 1) or not (labeled 0), and “*ChaSur*” refers to whether a character has a surrounding composition (labeled 1) or not (labeled 0). The more units in the lower structure hierarchy, the lower the repeatability and the weaker the complexity of the Chinese characters. We analyzed the complexity of the Chinese characters according to the number and types of logographemes, strokes, and segments. The variable “*NumChaStr*” referred to the number of strokes of a character, which was taken from the *Modern Dictionary of Common Characters in Chinese* (现代汉语通用字表; Chinese Ministry of Culture and State Language Affairs Commission, 1988). Linguists generally believe that there are about 30 kinds of strokes. Then, following Wang and Su (2012) and Wang et al. (2013), we further decomposed the stroke composition of the *Hanzi* to determine the “*NumChaSeg*,” referring to the number of segments in a character, the “*TypeChaStr*,” and the “*TypeChaSeg*,” referring to the number of stroke types and segment types of a character, respectively. For example, the character 右 (/you4/, right side) has five strokes (一, 丿, 丨, 𠃍, 一) and six segments (一, 丿, 丨, 一, 丨, 一) but just four stroke types and three segment types, as the stroke 𠃍 is decomposed into 一 and 丨. The variable “*NumLog*” refers to the number of logographemes in a character, which were counted according to the *Specification of Common Modern Chinese Character Components and Component Names* (现代常用字部件及部件名称规范; Ministry of Education of China, 2009).

#### Logographeme Complexity, Phonetic Identity, and Local Structure

We also determined the properties of the logographeme where the stroke is located. Using the same standards as above, “*NumLogStr*” and “*NumLogSeg*” refer to the number of strokes and segments, respectively, in the logographeme, and “*TypeLogStr*” and “*TypeLogSeg*” refer to the stroke types and segment types, respectively. Some logographemes are themselves Chinese characters (see the section on “The characteristics of Chinese characters” above) that have phonetic identities. Therefore, we used a categorical variable “*IsCha*” to refer to whether a logographeme has a phonetic identity (labeled 1) or not (labeled 0). For instance, the first stroke 一 and the third stroke 丨 of 右 are located in the first and the second logographemes, respectively, but the second logographeme 口 (/kou3/, mouth) can also be used as a *Hanzi* and has its own sound. Previous studies have shown that the component is likely to be the unit of orthographic representation (Han et al., 2007; Han and Bi, 2009; Chen and Cherng, 2013; Xu et al., 2018). In order to explore the breadth of “online” orthographic representation processing, we also mark the relevant attributes of the logographemes subsequent to the logographeme where the stroke is located. We used “*NumRestLog*” to represent the number of logographemes to be written behind the current logographeme. As the Chinese characters learned in this teaching stage only contain five logographemes at most, theoretically, it is only necessary to determine the information about the subsequent four logographemes at most for a stroke which is in the first logographeme. In the database, we used “*Back1*,” “*Back2*,” “*Back3*,” and “*Back4*” plus the variable names of the above five variables “*NumLogStr*,” “*NumLogSeg*,” “*TypeLogStr*,” “*TypeLogSeg*,” and “*IsCha*” to refer to the relevant attributes of the subsequent logographemes. For instance, when the writer writes any stroke in the current logographeme 木 in 楼 (/lou2/, building), the first logographeme subsequent to that stroke is 米 and the second is 女. Hence, “*Back2NumLogStr*” refers to the number of strokes of the second subsequent logographeme, 女. There is still a local and relative spatial relationship between each logographeme. Complex spatial relationships require a more precise coordinated operation of motor effectors, and more complex abstract motor coding needs to be retrieved (Schmidt and Wrisberg, 2000; Woch and Plamondon, 2004). For instance, 画 (/hua4/, painting) is a surrounding composition, but there are more complex relations locally among 一 ^①^, 田 ^②^, and 凵 ^③^ (the upper-right label on the logographeme represents the order in the writing execution). In detail, we should write 一 and 田 from top to bottom and then surround 田 with 凵. There is a larger movement distance between the offset of the last stroke of the logographeme in front and the onset of the initial stroke of the logographeme in the back compared with the writing strokes in the same logographeme. Hence, we used three categorical variables to label the local structure of logographemes through every initial stroke: “*LR*” refers to whether the logographeme is on the right side of the previous logographeme (labeled 1) or not (labeled 0), “*UD*” refers to whether the logographeme is under the previous logographeme (labeled 1) or not (labeled 0), “*Sur*” refers to whether the logographeme is surrounded by the previous logographeme (labeled 1) or not (labeled 0). Except for the initial stroke of each logographeme, other strokes were marked as 0 in all three variables.

#### Stroke Identity, Frequency, Complexity, Direction, and Position

With a closed stroke classification system and using a limited number of strokes to combine Chinese characters or logographemes, stroke repetition is bound to occur. For instance, 三 (/san1/, three) is a triple replication of the stroke 一, although there are differences among these three strokes in details such as length. We used a categorical variable “*IsRepeat*” to label whether the stroke is written repeatedly (labeled 1) or not (labeled 0). When the 3,000 Chinese characters in *Chinese Proficiency Grading Standards for International Chinese Language Education* are decomposed into strokes, it will be found that some strokes, such as 一, 丨, 丿, and 丶, are used very frequently. The frequency of occurrence of these four strokes together is as high as 74%. We used the statistical results in the study by Wang and Su (2012) regarding the frequency of occurrence and generativeness of strokes as two variables, “*Occurrence*” and “*Generative*,” which are not exactly the same and reflect the frequency factors of strokes. Generally, the more complex the strokes are, the lower their frequency of occurrence. We used two variables, “*NumSeg*” and “*TypeSeg*,” to define the stroke complexity with respect to the number and type of segments, following Wang et al. (2013). In addition, strokes that need to change direction multiple times in the writing process are obviously more difficult than strokes in a single direction (Woch and Plamondon, 2004). In particular, there are five forms of strokes of Chinese characters: horizontal (from left to right, e.g., 一), vertical (from top to bottom, e.g., 丨), diagonal (from top left to bottom right, or from top right to bottom left, e.g., 丿), arc (like an arc, e.g., 乚), and a mixture (mixing the above directions, e.g., ㄋ). As stroke direction is a categorical variable, it was dummy coded with five 1/0 binary variables: “*Horizontal*,” “*Vertical*,” “*Diagonal*,” “*Arc*,” and “*Mixture*,” where 1 indicates that it conforms to the form indicated by the variable name, and 0 otherwise. According to Kandel et al. (2006) and Zhang and Feng (2017), there are obvious interval time peaks among the graphemes (syllables or logographemes, also called a “boundary effect”) during handwriting, in which the velocity of the first stroke of the subsequent logographeme is slowest. Hence, the position of strokes was marked with three categorical variables: “*StartStr*” refers to whether a stroke is the initial stroke of the logographeme (labeled 1) or not (labeled 0), “*EndStr*” refers to whether a stroke is the last stroke of the logographeme (labeled 1) or not (labeled 0), and “*MiddleStr*” refers to whether a stroke is located between the initial and the last stroke of the logographeme (labeled 1) or not (labeled 0).

### The Analysis of the Orthographic Representation of Chinese Character Handwriting

We compiled the strokes and ISIs with 50 orthographic variables into a database. In this section, we report an analysis of the orthographic representation of Chinese character handwriting, making use of a large-scale handwriting database of CFL learners. To minimize individual differences in timing, we then transformed each ISI into z-scores (Z_ISI_). After deleting the aberrant ISI data (Z_ISI_ > 2), there were 12,951 valid stroke records left; Min. (ISI) = 0.024 s, Max. (ISI) = 4.814 s, Mean (ISI) = 0.450 s, SD (ISI) = 0.497 s.

Table 1 presents the descriptive results for the ISI and the orthographic variables, and Table 2 presents the correlations between the ISI and all other variables. From Table 2, we can see that (1) ISI was correlated with all character-located variables except *TypeChaStr*. It seems that the complexity of Chinese strokes and logographemes is negatively correlated with the ISI. Moreover, the ISI of the strokes in the left–right composition and surrounding composition was longer, and the ISI in the upper and lower composition was shorter. (2) ISI was correlated with all logographemic-located variables, except *NumLogStr*. The position of the stroke in a logographeme, whether the logographeme could form a character independently, and the complexity of the logographeme were significantly related to the ISI of the strokes. (3) There was a significant negative correlation with the three subsequent logographemes at most. (4) The ISI of strokes was related to the frequency and spatial form of the strokes being written. However, it had no significant relationship with the stroke segment complexity of the stroke itself.

**TABLE 1:** Descriptive statistics for all variables.

	*N*	Min	Max	Mean	SD
ISI	12,951	0.02400	4.81400	0.4497955	0.49727747
**Character-located variables**					
NumChaStr	12,951	4	16	8.63	2.444
NumLog	12,951	2	5	2.55	0.730
TypeChaStr	12,951	2	5	4.25	0.719
NumChaSeg	12,951	4	20	10.73	2.923
TypeChaSeg	12,951	2	5	4.07	0.704
ChaLR	12,951	0	1	0.53	0.499
ChaUD	12,951	0	1	0.28	0.447
ChaSur	12,951	0	1	0.18	0.388
**Logographemic-located variables**					
StartStr	12,951	0	1	0.21	0.404
MiddleStr	12,951	0	1	0.46	0.498
EndStr	12,951	0	1	0.34	0.473
LR	12,951	0	1	0.08	0.273
UD	12,951	0	1	0.09	0.292
Sur	12,951	0	1	0.03	0.172
NumLogStr	12,951	1	9	3.88	1.520
NumLogSeg	12,951	1	11	4.75	1.740
TypeLogStr	12,951	1	5	2.90	0.862
TypeLogSeg	12,951	1	5	2.93	0.909
IsCha	12,951	0	1	0.69	0.464
NumRestLog	12,951	0	4	0.64	0.775
**Logographeme-subsequent variables**					
Back1LogNumStr	12,951	0	8	1.57	1.886
Back1LogNumSeg	12,951	0	11	2.07	2.449
Back1LogTypeStr	12,951	0	5	1.25	1.435
Back1LogTypeSeg	12,951	0	5	1.36	1.570
Back1LogIsCha	12,951	0	1	0.33	0.469
Back2LogNumStrns	12,951	0	6	0.38	1.043
Back2LogNumSeg	12,951	0	7	0.50	1.356
Back2LogTypeStr	12,951	0	5	0.32	0.890
Back2LogTypeSeg	12,951	0	5	0.34	0.945
Back2LogIsCha	12,951	0	1	0.09	0.281
Back3LogNumStr	12,951	0	6	0.08	0.543
Back3LogNumSeg	12,951	0	7	0.10	0.652
Back3LogTypeStr	12,951	0	5	0.06	0.444
Back3LogTypeSeg	12,951	0	4	0.06	0.393
Back3LogIsCha	12,951	0	1	0.02	0.134
Back4LogNumStr	12,951	0	5	0.01	0.191
Back4LogNumSeg	12,951	0	5	0.01	0.195
Back4LogTypeStr	12,951	0	4	0.01	0.147
Back4LogTypeSeg	12,951	0	4	0.01	0.153
Back4LogIsCha	12,951	0	1	0.00	0.041
**Stroke variables**					
NumSeg	12,951	1	5	1.29	0.610
TypeSeg	12,951	1	4	1.27	0.560
Occurrence	12,951	0.012	27.107	15.65260	9.122566
Generative	12,951	0.021	16.812	12.45275	5.088043
Horizontal	12,951	0	1	0.30	0.460
Vertical	12,951	0	1	0.19	0.391
Diagonal	12,951	0	1	0.33	0.469
Mixtual	12,951	0	1	0.16	0.367
Arc	12,951	0	1	0.02	0.144
IsRepeat	12,951	0	1	0.42	0.494

**TABLE 2:** Correlations of all variables (*N* = 12,951).

ISI	Character-located variables											

	NumChaStr		NumLog	TypeChaStr	NumChaSeg		TypeChaSeg	ChaLR	ChaUD		ChaSur
	−0.063**		−0.041**	−0.005	−0.046**		0.020*	0.018*	−0.038**		0.020*

	**Logographeme-located variables**											

	StartStr	MiddleStr	EndStr	LR	UD	Sur	NumLogStr	NumLogSeg	TypeLogStr	TypeLogSeg	IsCha

	0.258**	−0.089**	−0.126**	0.201**	0.130**	0.067**	−0.011	0.020*	−0.020*	0.093**	−0.028**

	**Logographeme-subsequent variables**											

	NumRestLog	Back1Log-NumStr	Back1Log-NumSeg	Back1Log-TypeStr	Back1Log-TypeSeg	Back1Log-IsCha	Back2Log-NumStrns	Back2Log-NumSeg	Back2Log-TypeStr	Back2Log-TypeSeg	Back2Log-IsCha

	−0.060**	−0.061**	−0.048**	−0.048**	−0.040**	−0.051**	−0.050**	−0.053**	−0.052**	−0.057**	−0.037**

	Back3Log-NumStr	Back3Log-NumSeg	Back3Log-TypeStr	Back3Log-TypeSeg	Back3Log-IsCha	Back4Log-NumStr	Back4Log-NumSeg	Back4Log-TypeStr	Back4Log-TypeSeg	Back4LogIsCha	

	−0.029**	−0.031**	−0.026**	−0.030**	−0.022*	−0.007	−0.008	−0.004	−0.006	−0.003	

	**Stroke variables**											

	NumSeg	TypeSeg	Occurrence	Generative	Horizontal	Vertical	Diagonal	Mixtual	Arc	IsRepeat	

	−0.004	−0.008	0.019*	0.026**	−0.027**	0.017*	0.036**	−0.035**	0.014	−0.034**	

Symbol * indicates significance at the 0.05 level. Symbol ** indicates significance at the 0.01 level.

Because there were correlations among the predictors (Table 2), before conducting regression analyses, we checked for collinearity issues using a stepwise variance inflation factor (VIF) selection procedure with a wrapper of the VIF function in the fmsb R package (for the stepwise VIF selection procedure, see^1^). Setting the VIF threshold at 5, we found that having more than 20 variables led to collinearity. For instance, in the case of *NumSeg* and *Mixture*, strokes in multiple directions must be composed of more than two segments. To address this, we removed 22 variables. Further stepwise VIF selection revealed no collinearity among the remaining predictors. Hence, in the subsequent regression analyses, we included the following predictors, as shown in Table 3.

**TABLE 3:** The predictor variables in the regression analyses.

Character located	Logographeme-located	Logographeme(s)-subsequent	Stroke
NumLog	EndStr	Back1LogNumStr	Generative
TypeChaStr	LR	Back1LogTypeSeg	Vertical
NumChaSeg	UD	Back1LogIsCha	Diagonal
TypeChaSeg	Sur	Back2LogTypeSeg	Mixtual
ChaUD	NumLogStr	Back2LogIsCha	Arc
ChaSur	TypeLogStr	Back3LogTypeSeg	IsRepeat
	TypeLogSeg	Back3LogIsCha	
	IsCha	Back4LogTypeSeg	

The results of the regression model (*R*^2^ = 0.102, adjusted *R*^2^ = 0.100) are reported in Table 4. Assuming that the ISI reflects the retrieval of orthographic programming codes, the orthographic representation in “online” processing is multilevel, including the whole character, logograms, and the strokes themselves. **The variables of the logographeme where the stroke is located affect the ISI of strokes:** the position of the stroke in the logographeme has a significant impact on the ISI of the stroke. Whether the logographeme to be written is on the right, below, or surrounded by the previous one, the ISI before the starting stroke of the logographeme is longer than those of the strokes at the middle and end of the logographeme. The stroke at the end of the logographeme shows the opposite trend, however. Hence, there is an obvious logographemic boundary effect, as the ISI is longer, with more strokes and more types of segments. Surprisingly, however, the shorter the ISI, the fewer stroke types in the logographeme, which may be related to the mapping relationship between strokes and segments. In addition, the ISI of strokes in a component that can form a character independently on other occasions and has its own phonetic identity is shorter. **The variables of the subsequent logographeme also impact the ISI of strokes in the logographeme being written:** the ISI is longer, with fewer strokes but more segment types, in the subsequent logographeme. The identity of the subsequent logographeme (whether it can form a character independently or not) has a marginally significant impact on the ISI of strokes in the logographeme being written. Interestingly, the breadth of online orthographic representation processing is not limited to the logographeme being written but cascades over the first subsequent logographeme. In addition to the logographeme level, **ISI is also affected by several variables of the whole character and stroke itself.** The ISI is longer, with fewer segments (types) or with more strokes of character. Compared with other compositions, the ISI of strokes in up–down compositions tends to be shorter. The ISI of repeated strokes is shorter, whereas the ISI of more generative strokes is shorter, indicating that the information of orthographic representation includes the complexity, structure, and stroke identity of Chinese characters.

**TABLE 4:** Results of regression on ISI.

	*B*	*t*	*p*
Intercept	0.291	6.577	<0.000
NumLog	−0.015	−1.588	0.112
**TypeChaStr**	**0.042**	**5.207**	**<0.000**
**NumChaSeg**	**−0.008**	**−3.195**	**0.001**
**TypeChaSeg**	**−0.028**	**−3.474**	**<0.000**
**EndStr**	**−0.037**	**−3.321**	**<0.000**
**LR**	**0.378**	**22.403**	**<0.000**
**UD**	**0.268**	**16.336**	**<0.000**
**Sur**	**0.265**	**10.133**	**<0.000**
**ChaUD**	**−0.021**	**−1.989**	**0.047**
ChaSur	0.019	1.543	0.123
**Back1LogNumStr**	**−0.020**	**−4.056**	**<0.000**
**Back1LogTypeSeg**	**0.037**	**6.695**	**<0.000**
Back1LogIsCha	−0.022	−1.714	0.087
Back2LogTypeSeg	−0.009	−1.246	0.213
Back2LogIsCha	0.002	0.08	0.936
Back3LogTypeSeg	−0.018	−0.902	0.367
Back3LogIsCha	0.074	1.233	0.218
Back4LogTypeSeg	0.017	0.598	0.55
**NumLogStr**	**0.021**	**4.282**	**<0.000**
**TypeLogStr**	**−0.085**	**−9.292**	**<0.000**
**TypeLogSeg**	**0.101**	**13.801**	**<0.000**
**IsCha**	**−0.031**	**−3.184**	**0.001**
**Generative**	**0.005**	**3.846**	**<0.000**
Vertical	0.008	0.651	0.515
Diagonal	0.018	1.563	0.118
Mixtual	−0.010	−0.518	0.604
Arc	0.017	0.482	0.63
**IsRepeat**	**−0.056**	**−5.535**	**<0.000**

*Bold values highlight statistically significant independent variables and their values.*

**FIGURE 4:**
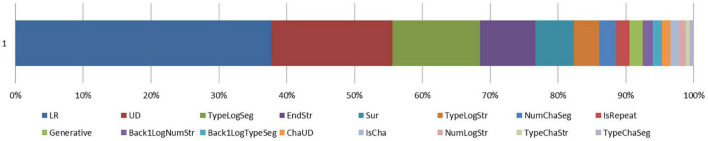
Relative contributions of the different predictors to the ISI.

To gain a better understanding of the relative importance of the predictors, we also used the *calc.relimp* function in the *relaimpo* R package to determine the relative importance of a predictor in the face of other predictors (Johnson and Lebreton, 2004; Grömping, 2006). Initially, we used three variables (*StartStr*, *MiddleStr*, *EndStr*) to represent the position of the stroke in the current logographeme. In order to describe the relative position between logographemes in detail, we assigned the relative position attribute to the initial stroke of the current logographeme. Therefore, the strokes with three variable (*LR*, *UD*, *Sur*) values of 1 were essentially the initial strokes; that is, *StartStr* = *LR* + *UD* + *Sur*. As shown in Figure 4, for ISI (as a measure of “online” orthographic access), the most important variables were those related to the position of the stroke: “*LR*,” “*UD*,” “*Sur*,” and “*EndStr*,” which explained more than 69% of the variance. The complexity of the logographeme where the stroke is located was also a key determinant of ISI, and the variables *TypeLogSeg*, *TypeLogStr*, and *NumLogStr* explained about 18% of the variance. In other words, the seven above-mentioned variables related to the logographeme located could explain more than 88% of the variance. The explanatory power of the variables from the subsequent logographeme, whole character, or stroke itself was relatively small and could explain 2.8, 5, and 3.8% of the variance, respectively. It can be said that the orthographic representation of real-time processing is multilevel, but that logographemes play a very important role, probably working as units of representation, as expected.

## Discussion

The current study represents the first systematic and large-scale empirical investigation into the orthographic representation of *Hanzi* by CFL learners during the execution of handwriting movements. In the field of written production, to explore how orthography may be represented and organized, many studies have used writing latency as the dependent variable to uncover the planning process before writing execution (Chen and Cherng, 2013; Damian and Qu, 2017; Wang et al., 2019). The second approach to exploring the cognitive mechanisms underlying writing production is to analyze the phenomenon of handwriting output errors in specific types of brain injury cases (Law and Leung, 2000; Han et al., 2007; Han and Bi, 2009). Another approach investigated the process of writing execution from a motor perspective (van Galen, 1991), and these studies utilized inter-letter/stroke interval or stroke velocity in writing execution as the dependent variables to explain the movement processes involved in written word production (Kandel et al., 2006; Vilageliu and Kandel, 2012; Zhang and Feng, 2017).

### The Hierarchy and Unit of Orthographic Representation

In this study, following the third approach, we tried to study how orthography is represented and organized, taking the ISI as an index of parallel processing of multilevel orthographic representation, together with 50 orthographic variables from the whole character, logographeme, and stroke. After excluding 22 variables that caused collinearity, we used the 28 remaining independent variables as predictors to conduct the regression analyses. The results showed that orthographic representation has a hierarchy and that different representational levels are active simultaneously at the lower levels of van Galen’s (1991) model. Although properties from logographemes (the current logographeme and the subsequent one) accounted for 91.2% of the variance, those from the whole character and stroke to be written still have a certain explanatory power. At the whole character level, complexity and composition accounted for 4.9% of the variance; at the stroke level, identity and generativeness accounted for 3.9% of the variance, which is compatible with the findings of Han et al. (2007) and Lo et al. (2016). This result partially supported Houghton and Zorzi’s (2003) dual-route model, which proposed two distinct representational levels, one for grapheme units and another for letter units. However, our findings differ from the dual-route model in suggesting that there may be a representation level of the whole character in *Hanzi* writing, which may be related to the complexity of the structure of Chinese characters. Among all predictors, logographemic properties play a decisive role, which is consistent with the findings of Han et al. (2007), indicating that the logographeme is likely to be an orthographic unit of representation. The more complex the logographeme (more strokes, more segment types), the longer the ISI. Moreover, the ISI of strokes in logographemes with phonetic identity is significantly shorter than that of logographemes without phonetic identity, indicating that the phonetic identity or the integrity of the logographeme can make orthographic representation easier to access, which is compatible with the findings of Han and Bi (2009). Our study has revealed more information about orthographic representation, but how different levels of representation operate and relate to each other—in other words, whether the representation at the three levels of whole character, logographeme, and stroke is processed in parallel or in sequence—needs to be studied further.

### The Commonalities and Differences in the Orthographic Representations of Writing and Reading

The stroke is a structural unit that affects Chinese character recognition. Several studies have investigated the effects of the number, self-complexity, repeatability, and position of strokes on Chinese character recognition (Flores d’Arcais, 1994; Peng and Wang, 1997; Zhang et al., 2002; Wang et al., 2003). These results showed that the reaction time for *Hanzi* with more strokes is longer than that for *Hanzi* with fewer strokes and that the recognition of *Hanzi* composed of repeated strokes is faster and the error rate is lower than for *Hanzi* composed of non-repeated strokes. Moreover, different stroke positions have different effects on *Hanzi* recognition. Generally speaking, the initial stroke plays a more important role in recognition than the end stroke. In our study, the ISI of writing repeated strokes is shorter and the ISI of initial strokes is longer than that of middle and end strokes, which is a commonality in the orthographic representation of the writing and reading of *Hanzi*. Surprisingly, we found that the number of strokes had the opposite effect on writing and recognition. In the regression analysis, variables related to the number and type of strokes/segments could explain 24% of the variance. However, most of these variables were significantly negatively correlated with the ISI, which made the content of orthographic representation in recognition and writing different in terms of strokes.

At the logographeme level, the attributes of the current logographeme and, at most, three subsequent logographemes were significantly correlated with the ISI of the stroke, and properties from logographemes (the current logographeme and the subsequent one) could account for 91.2% of the variance, indicating that the number of logographemes was an important element of the orthographic representation, which is consistent with the conclusions of *Hanzi* recognition research (Peng and Wang, 1997). Moreover, the present study also found that orthographic representation also includes the complexity of the logographeme itself, such as the number and type of strokes/segments. In addition, both *Hanzi* recognition and writing contain a representation of the logographeme’s relative position. However, in this study, the ISI of the stroke in the right logographeme was longer than that in the lower or surrounding logographeme, contrary to the conclusions of Yu et al. (1990), which may be related to the experimental task. Our study adopted the direct copying task, whereas their research named the logographemes under the condition of the whole character.

At the whole character level, CFL learners find it easier to decompose left–right compositions (Hao and Fan, 2008). The conclusions of our study also support the representation of Chinese character structure in writing, but the results showed that the ISI of strokes in up–down compositions was shorter than in other compositions, contrary to the findings of Hao and Fan (2008). Hao and Fan (2008) used new characters composed of familiar logographemes, a method completely different from that of our study in terms of the familiarity of the experimental materials. Perhaps, for unfamiliar *Hanzi*, CFL learners tend to segment Chinese characters in the usual left–right linear direction, whereas up–down decomposition is more advantageous for familiar *Hanzi*.

### The Similarities and Dissimilarities in Orthographic Representation Between Native Chinese Writers and Chinese as a Foreign Language Learners

Compared with the research on CFL learners, there are relatively more studies on the orthographic representation of Chinese characters handwritten by Chinese native writers, including patients with brain injuries and healthy writers. The logographeme is likely to be an orthographic unit of representation; in another way, there is a logographeme boundary effect in Chinese characters during writing execution, which is compatible with the findings for CFL learners (Xu et al., 2018) and native writers (Han et al., 2007; Chen and Cherng, 2013; Zhang and Feng, 2017). In CFL learner-oriented research, Xu et al. (2018) found that there was no significant difference in the interval effect of critical strokes between up–down and left–right characters. However, our findings differ from the results of Xu et al. (2018) in suggesting that the ISI of strokes in up–down compositions is shorter than in other compositions. In native writer-oriented research, orthographic representation includes not only the attributes of the logographeme, such as the (phonetic) identity (Han et al., 2007; Han and Bi, 2009) and complexity (Zhang and Feng, 2017), but also that of the stroke, such as the stroke identity (Lo et al., 2016). Our results show that the conclusions of previous studies on native writers are also applicable to CFL learners, meaning that there are similarities between the two. Moreover, the present research found that the orthographic representation of CFL learners was a hierarchy on the three levels of the whole character, logographeme, and stroke. This study also extensively analyzed the details of orthographic representation and found more information, such as the relative position of the logographeme, the repeatability of the stroke, the number of stroke types in the logographemes, and the attributes identified by segments, which was beneficial to the use of the pedagogical corpus. In addition, there are also differences between native writers and CFL learners in the occurrence and breadth of online processing of logographemes. See the following for details.

### Occurrence of Online Processing of Logographemes

Most studies of the orthographic representation of *Hanzi* handwriting to date have revealed that logographemes are units of orthographic representation in handwriting production (Law and Leung, 2000; Han et al., 2007; Chen and Cherng, 2013). We conclude that in the multilevel orthographic structure of representation, the representation of the logographeme is absolutely dominant, which is basically consistent with the findings of previous studies. During handwriting movement, there is obviously a logographeme boundary effect (Zhang and Feng, 2017; Xu et al., 2018), like the syllable boundary effect in alphabetic languages (Kandel et al., 2006, 2009; Kandel and Valdois, 2007; Vilageliu and Kandel, 2012). In the regression analysis, the variable *StartStr* was eliminated on the grounds of collinearity, but the three relative position variables (*LR*, *UD*, *Sur*) entered the regression analysis and had a significant predictive effect (accounting for more than 60% of the variance) on ISI, as expected. The ISI before the initial stroke of the current logographeme is longer, whereas the ISI time of the end stroke of the current logographeme is shorter than that of other positions, indicating that, like native speakers, an obvious logographeme boundary effect in the process of handwriting *Hanzi* is also to be found among CFL learners.

It is generally believed that the abstract coding of the first logographeme is activated before writing execution, and other logographemes are processed online in parallel during execution. Zhang and Feng (2017) showed that for Chinese characters with higher frequency and fewer strokes in the first logographeme, the online processing for the second logographeme occurs at the end stroke of the initial logographeme, whereas for Chinese characters with low frequency and more strokes in the initial logographeme, the processing is delayed to the initial stroke of the second logographeme. In CFL teaching, we usually teach Chinese characters with high frequency and generativeness. Frequency is a meaningful influencing factor for native speakers, but for a CFL beginner, frequency may not play a major role. Therefore, there was a significant ISI decrease at the end stroke of the first logographeme due to the absence of planning for the second logographeme.

### Breadth of Online Planning of Logographemes

As mentioned earlier, previous studies have shown that logographemes play a key role in the representation of writing orthography and the second logographeme of Chinese characters is processed in parallel during the writing of the initial logographeme because processing capacities are limited in writing latency. However, there is no clear research on the extent to which online cognitive processing cascades to subsequent logographemes, as the writing materials in many experiments only contained composite characters with two logographemes (Zhang and Feng, 2017; Xu et al., 2018). In our database, we collected a larger collection of character types, including 134 two-logographeme character types, 69 three-logographeme character types, 24 four-logographeme character types, and 5 five-logographeme character types, which made it possible to understand the breadth of online processing in logographeme units. According to the correlation, as shown in Table 2, we can see that the ISI of the stroke in the initial logographeme was related to the attributes of the subsequent three logographemes in the most complex five-logographeme characters. The results of the regression analysis showed that the current logographeme where the stroke was located could explain about 88% of the variance and that the first subsequent logographeme could explain about 3% of the variance. Both showed that the proportion of orthographic representation for logographemes reached about 91%. In other words, the online processing of the current logographeme in the composite character mainly occurs in the ISI of the initial stroke of the current logographeme, but the processing for the current logographeme has probably been done with a very small contribution when writing the previous logographeme. Hence, although there are more than two logographemes in a *Hanzi*, the online processing in a logographeme unit can only cascade to the first subsequent logographeme, and any effect of further ones is undetectable.

### New Chinese Character Orthographic Units and Complexity Indexes

As described in the section on “The characteristics of Chinese characters,” linguists generally analyze a character in terms of three levels: radical, logographeme, and stroke. In addition, in the field of computer Chinese character recognition, the segment is the most basic graphic unit. Although only one study has shown that the number of segments affects orthographic access to Chinese characters (Wang et al., 2013), researchers have established many interpretative models for simple writing motion from the perspective of motion control (Plamondon, 1995; Grossberg, 2013). Writing a simple stroke with only one segment is an impulse response process, and writing a composite stroke with multiple segments is a linear combination of multiple impulse response processes. The complexity of writing motion execution increases as the number of segments increases. To our knowledge, most existing studies of the handwriting production of Chinese characters have used the number of strokes or components (radicals or logographemes) to define the complexity of orthography and have explored the effect of strokes and components on the access of orthography and writing execution (Lo et al., 2016; Wang et al., 2019). Compared with alphabetic languages, the separability of Chinese characters into strokes allows us to define the complexity of Chinese characters with less granular structural units. In the present study, those variables defined by segment could account for 17% of the variance, whereas the explanatory power of those variables defined by stroke was weaker, about 7%, which means that the segment may be a more effective structural unit for describing orthographic complexity. Interestingly, the effects of strokes and segments on orthographic processing during handwriting movement are usually not the same and are sometimes even opposite. For instance, the number and type of segments of the logographeme are significantly positively correlated with the ISI, whereas the number and type of strokes are significantly negatively correlated with the ISI, which may be related to the characteristics of the configuration system of Chinese characters. Although there are more than 20,000 Chinese characters, there are only about 32 kinds of strokes and 8 kinds of segments. Taking 口 as an example, it has three strokes, three stroke types, but four segments and two segment types. Characters with more stroke types may have fewer stroke segment types, which is particularly obvious in Chinese characters with symmetrical structures.

## Conclusion

The present study made a first systematic analysis on the orthographic representation based on a large-scale pedagogical corpus of handwriting responses by CFL learners to a large sample of characters. The study used inter-stroke interval (ISI) as the index of parallel processing of multilevel orthographic representation from a motor perspective and made a mega-analysis on the depth and breadth of orthographic representation. We showed that orthographic representation has a hierarchy and that the logographeme is absolutely dominant, although three representational levels are active simultaneously during writing execution. Except for the first logographeme, the online processing of the logographeme to be written in the composite character mainly occurs before the initial stroke of the current logographeme, and the processing for the current logographeme has been done with a very small contribution when writing the previous logographeme. In addition, there exit the commonalities and differences between in the orthographic representations of writing and reading. Finally, we found that the segment is a more effective structural unit for describing orthographic complexity. These findings help us gain a deeper understanding of the cognitive mechanisms underlying writing and Chinese character recognition in reading and provide enlightenment on how to carry out effective Chinese character writing teaching in CFL.

## Data Availability Statement

The original contributions presented in the study are included in the article/Supplementary Material, further inquiries can be directed to the corresponding author.

## Author Contributions

JZ independently designed experiments, analyzed data, and wrote the manuscript.

## Conflict of Interest

The author declares that the research was conducted in the absence of any commercial or financial relationships that could be construed as a potential conflict of interest.

## Publisher’s Note

All claims expressed in this article are solely those of the authors and do not necessarily represent those of their affiliated organizations, or those of the publisher, the editors and the reviewers. Any product that may be evaluated in this article, or claim that may be made by its manufacturer, is not guaranteed or endorsed by the publisher.

## Footnotes

1
https://www.r-bloggers.com/collinearity-and-stepwise-vif-selection
